# A Case Report: Acute Myeloid Leukemia (FAB M7)

**Published:** 2014-12-10

**Authors:** R Masoumi-Dehshiri, AS Hashemi, H Neamatzadeh, H Zare-Zardeini

**Affiliations:** 1 Nutrition and Food Security Research Centre, Shahid Sadoughi University of Medical Sciences, Yazd, Iran; 2 Hematology and oncology Research Center, Shahid Sadoughi University of Medical Sciences and Health Services, Yazd, Iran.

**Keywords:** acute myeloid leukemia, bone marrow examination, weakness

## Abstract

Acute myeloid leukemia (AML-M7) is a type of pediatric AML accounting for 3–10% of primary childhood AML and children may present with a broad variety of symptoms including low-grade fever, diarrhea, easy bruising, failure to gain weight and life-threatening conditions. We report a rare case of AML .A 26-month-old boy who presented with weakness and fatigue. He was diagnosed as a case of AMLM-7 on the basis of peripheral blood finding, bone marrow examination report and immune phenotyping.

## Introduction

Acute myeloid leukemia (AML) is the most common acute leukemia in adults [,]. In the United States and Europe, the incidence has been stable at 3 to 5 cases per 100,000 populations [-]. In contrast, AML accounts for less than 10 percent of acute leukemia's in children less than 10 years of age.AML comprises a type of hematologic malignancies with variable outcomes and characterized by a clonal proliferation of myeloid precursors with a reduced capacity to differentiate into more mature cellular elements. As a result, there is an accumulation of leukemic blasts or immature forms in the bone marrow, peripheral blood, and occasionally in other tissues, with a variable reduction in the production of normal red blood cells, platelets, and mature granulocytes (5)

The un-differentiated myeloid cells show chromosomal abnormalities in about 55% of cases of adult AML [6]. Translocations are used for disease classification [6,7]. Although a presumptive diagnosis of AML can be made via examination of the peripheral blood smear when there are circulating leukemic blasts, a definitive diagnosis usually requires an adequate bone marrow aspiration. A portion of the biopsy can be submitted in saline or preferably culture medium and crushed in the flow cytometry laboratory to isolate a blast cell suspension for analysis [8].

The French-American-British classification (FAB) Classification sub-types of AML based on morphology and cytochemical staining with immunophenotypic data in some instances. Types (M0, M1, M2, and M3) are predominantly granulocytic and differ according to the extent of maturation. M4 is both granulocytic and monocytic, with at least 20%monocytic cells, whereas M5 is predominantly monocytic (at least 50% monocytic cells). M6 shows primarily differentiation with dysplastic features including megaloblastic changes, M7 is acute megakaryocytic leukemia (AML-M7) identified by the presence of megakaryocytic antigens demonstrated by flow cytometry or immunohistochemistry or the presence of platelet peroxides [9].

## Case report

A 26-month-old boy patient from Iran who was admitted 9 month ago to the shahid sadoughi hospital that presenting with neutropenia associated with anemia and thrombocytopenia. There was no history of any hematological disorder. On examination; the patient had pallor and splenic enlargement, measuring 23 cm in ultrasonography.

Physical examination was otherwise unremarkable. The patient had been suffering from recurrent febrile episodes and nocturnal sweats with weakness and fatigue. Morphology and Immunophenotyping Peripheral blood cells were examined by an automated hematologic analyzer (Sysmex, XE-5000, Vienna, Austria).

Peripheral blood smear examination showed normocytic normochromic red blood cells including few nucleated red blood cells, white blood cells showed left, shift with significant number ofblast that suggestive of acute leukemia.Many giant platelets and platelet aggregates were seen. The leukocyte differential count was eosinophils5%, lymphocytes 62%, and neutrophils 31%and band forms 1%. Coagulation tests showed a prolonged prothrombin time of 16.3 sec (reference range, 10.2 to 13.8), a normal activated partial thromboplastin time, a normal fibrinogen and an increased D-dimer concentration of 4.96 mg/mL (reference range, 0 to 0.35). Bone marrow smears were stained with Wright-Giemsa and analyzed according to routine clinical laboratory procedures. Bone marrow aspiration and biopsy showed increased abnormal megakaryocytic, Monolobated and multinucleated megakaryocytic with hyper chromatic and pleomorphic nuclei were seen and showed the leukemic cells were positive for CD13 , CD33, CD42 and CD61 and negative for CD3, CD5, CD7, CD20, CD22 and human leukocyte antigen-DR. The biochemical parameters such as uric acid, bilirubin, creatinine, liver enzymes were normal. Serum LDH was slightly raised. The diagnosis was confirmed as AML-M7 as the blasts were positivefor CD42 and CD61 (megakaryocyte specific antigen) along with myeloid markers CD13 and CD33. Based on this diagnosis and with respect to the patient’s severely compromised overall condition, therapy withal-trans retinoic acid (ATRA, 10 mg/kg) was initiated, followed by cytarabine- and anthracycline-based induction polychemotherapy after 5 days and died four months after diagnosis.

## Discussion

The incidence of acute leukemia is approximately 2.3 per 100000 people per year.AML M-7 is a rare subtype of leukemia and represents 1.2% of cases of adult leukemia, compared to 3-10% of childhood leukemia (10).It is classified under M-7 in the French-American-British classification (11).

The patient was a 26 month years old male. Clinical features are not different from other type of AML but organomegaly is noted infrequently in adults. In our patient symptoms of anemia that is progressive weakness and diabetic were symptoms. Cytopenias are usually present but 30% of patients have platelet counts >100000/uL but in our case who had low platelet count (40x109/L). Osteosclerotic and osteolytic lesions have been described in few case reports [12, 13, 14].

The diagnosis depends on the expression of at least one platelet antigen (CD41, CD42b, and CD61) on the leukemic cells [13, 14].In our patient bone marrow aspiration was done that showed increased abnormal megakaryocytic, Monolobated and multinucleated megakaryocytic with hyper chromatic and pleomorphic nuclei were seen and showed the leukemic cells were positive for CD13, D33,CD42 and CD61 and negative for CD3, CD5, CD7, CD20, CD22, and human leukocyte antigen-DR.Cytogenetic analysis was not carried out in this case. AML M 7 may present as de novo leukemia, secondary leukemia after chemotherapy, or transformed myeloproliferative disorders and myelodysplastic syndromes [15]. This report describes a presentation of AML M7 with thrombocytopenia. Although, nonspecific, cytogenetic abnormalities are more frequent (>90%) in AMLM-7 than in other subtypes of AML (14). The prognosis is significantly poor in AML M7 with megakaryocytic blast crisis. AML M7 by itself is an adverse prognostic factor for disease-free survival. However remission and long term survival are common in children with AML M7 specially in children with Down syndrome (15,16). This patient had partial recovery and clinically stable for several days with symptomatic treatment despite poor prognosis of AML-M7 and died four months after diagnosis.

## Conclusion

AML M7 is a rare manifestation, patients with acute megakaryoblastic leukemia validated by a review of morphologic and immunophenotyping data is the largest comprehensive series with cytogenetic data to assess their frequencies and to emphasize the differences between M7 developing in infancy and adulthood. We conclude acute megakaryocytic leukemia should be considered in the differential diagnosis of undifferentiated acute leukemia with low platelet count.
